# Social participation and physical prefrailty in older Japanese adults: The Shimane CoHRE study

**DOI:** 10.1371/journal.pone.0243548

**Published:** 2020-12-16

**Authors:** Takafumi Abe, Kenta Okuyama, Masamitsu Kamada, Shozo Yano, Yuta Toyama, Minoru Isomura, Toru Nabika, Naoki Sakane, Hitoshi Ando, Ryo Miyazaki

**Affiliations:** 1 Center for Community-Based Healthcare Research and Education (CoHRE), Organization for Research and Academic Information, Shimane University, Izumo City, Shimane, Japan; 2 Center for Primary Health Care Research, Lund University, Malmö, Sweden; 3 Department of Health and Social Behavior, School of Public Health, Graduate School of Medicine, The University of Tokyo, Bunkyo-ku, Tokyo, Japan; 4 Department of Laboratory Medicine, Faculty of Medicine, Shimane University, Izumo City, Shimane, Japan; 5 Faculty of Human Sciences, Shimane University, Matsue City, Shimane, Japan; 6 Department of Functional Pathology, Faculty of Medicine, Shimane University, Izumo City, Shimane, Japan; 7 Division of Preventive Medicine, Clinical Research Institute for Endocrine and Metabolic Disease, National Hospital Organization, Kyoto Medical Center, Fushimi-ku, Kyoto, Japan; 8 Department of Cellular and Molecular Function Analysis, Kanazawa University Graduate School of Medical Sciences, Kanazawa City, Ishikawa, Japan; Ehime University Graduate School of Medicine, JAPAN

## Abstract

As older adults in an early stage (prefrailty) of frailty may return to a healthy state, it is necessary to examine the prevention of prefrailty. In this context, the number and types of social participation activities associated with physical prefrailty in community-dwelling older adults have remained relatively unexplored. This cross-sectional study investigates this issue by analyzing 616 participants living in Okinoshima, Shimane, a rural area of Japan, in 2019. Frailty was assessed using the 5-item frailty phenotype (unintentional weight loss, self-reported exhaustion, weakness, slow walking speed, and low physical activity). Data on social participation were obtained using a questionnaire based on participants’ level of involvement with volunteer groups, sports clubs/groups, neighborhood associations, religious organizations/groups, and community elderly salons; their answers were categorized as “yes” if they answered “several times per year or more” and “no” if they answered “never.” Binominal logistic regression was used to estimate odds ratios (OR) and 95% confidence intervals (CI) of prefrailty by the number or types of social participation activities, adjusted for gender, age, body mass index, smoking, medication-taking, educational attainment, working status, and living arrangement. Of the 616 participants, 273 (44.3%) and 28 (4.5%) had prefrailty and frailty, respectively. The analysis showed that the number of social participation activities was significantly associated with lower odds of prefrailty (OR = 0.83; 95% CI, 0.74–0.94). Regarding the types of social participation, sports clubs/groups were associated with lower odds of prefrailty (OR = 0.47; 95% CI, 0.31–0.73). Participation in neighborhood associations was associated with prefrailty/frailty (OR = 0.57; 95% CI, 0.37–0.86). These results suggest that increasing the number of social participation activities or involvement in sports clubs/groups and neighborhood associations may be important to prevent physical prefrailty in the older population.

## Introduction

Frailty is a major symptom of age-related decline in late life. It is defined as a state of heightened vulnerability to stressors as a result of reduced physiological reserve due to a cumulative decline in the ability to maintain homeostasis and impairments in multiple systems [1]. Physical frailty is a risk factor for a poor quality of life, physical disability, and mortality [2–4]. Previous reviews have reported a global prevalence of frailty ranging from 4.9% to 27.3% [5–7]. A study reported that the prevalence rates of prefrailty and frailty in older Japanese adults aged 65 years or older were 48.1% and 7.4%, respectively [8]. Therefore, the prevention of frailty is an important public health concern [9].

Recently, the concept of frailty has been expanded beyond its physical aspect to include psychological and social aspects. In a scoping review of social frailty, social participation was suggested as a component of social behavior or activities [10]. Moreover, Duppen et al.’s systematic review proposed that social participation, one of the elements of the social environment, be modified to include the frailty concept [11]. The studies included in these reviews [10, 11] also reported that social participation is a predictor of frailty. The SHARE study, which included 11 European countries, reported a significant association between the deterioration of frailty and the lack of social participation, which included engaging in voluntary work, caring for sick persons, or participating in sports clubs [12]. Recently, a longitudinal study found that improvements in the frailty status of older Japanese adults were affected by regular participation in any of the eight included organization types [13]. Meanwhile, Woo et al.’s cross-sectional study reported that participation in community or religious activities was associated with lower frailty scores in older women [14]. On the contrary, some studies reported that the number of social participation activities was negatively associated with frailty among older adults [15, 16]. However, these studies did not investigate the association between both the number and types of social participation activities and physical frailty [10–16]. Social participation may depend on social resources in a residential area, such as differences between urban and rural areas [17]. Clarifying the relationship between the status of social participation and physical frailty in the rural region is important to promote frailty prevention measures among older adults. In particular, interventional action is recommended for community-dwelling older adults with prefrailty status for the prevention of transition to frailty. We hypothesized that social participation affects the transition to prefrailty among older adults. The present study aimed to investigate the number and types of social participation activities negatively associated with physical prefrailty among community-dwelling older Japanese adults.

## Materials and methods

### Study design

This cross-sectional study was part of the Shimane CoHRE Study conducted by the Center for Community-Based Healthcare Research and Education, Shimane University. Japanese people with national health insurance are eligible for a health examination in their municipality of residence. The data for this study were collected from a health examination conducted in Okinoshima town (population: 14608; aging rate: 38.4%; area: 242.8 km^2^ (based on data from Japan’s census in 2015)), Shimane, rural Japan, in June 2019. Overall, 666 older adults participated in health examinations. The present study invited residents to voluntarily participate in the survey and did not provide any incentive. Participant inclusion criteria were: participated in a health examination, agreed to respond to our survey, and availability of data about frailty. After excluding participants with non-responses to the survey (n = 6) and missing data about frailty (n = 44), we analyzed data from 616 participants (response rate, 99.1%). The study protocol was approved by the Ethics Committee at Shimane University (#2888), and written informed consent was obtained from all participants before enrollment.

### Physical frailty

We considered the physical frailty phenotype as being characterized by the presence of three or more of the following five conditions, based on Fried et al. and modified for the Japanese population according to the Japanese version of the Cardiovascular Health Study (J-CHS) criteria [5, 18]: slowness, weakness, exhaustion, low activity levels, and weight loss. Participants who did not have any of these conditions were considered non-frail, whereas those reporting one or two of these conditions were considered prefrail.

Slowness and weakness were assessed by objectively measuring gait speed and grip strength. Gait speed was measured using a sheet-type pressure sensor (45.5 × 183 cm plate sensor; Healthwalk; Kao Corporation, Japan) placed in the center of a 5-meter walkway [19]. Participants were asked to walk freely along a 7-meter course and return to the starting point. The average walking speed to complete the distance for both right and left feet was used for the analysis. Slow gait speed was determined based on a cutoff speed of < 1.0 m/s [20]. Grip strength was measured using a Smedley-type handheld dynamometer (GRIPD; Takei, Niigata, Japan). Weakness was identified using maximum grip strength, and sex-specific cutoffs were applied (< 26 kg for men and < 18 kg for women) [21]. Exhaustion was identified based on a “yes” response to the following question: “In the last two weeks, have you felt tired for no reason?” Participation in physical activity was measured by the following items: (1) “Do you engage in regular physical exercise or sports to improve your health (including agricultural activities)?” and (2) “Do you engage in light-intensity levels of physical exercise to improve your health?” If participants answered “no” to both questions, we categorized them into the low-physical activity group in accordance with a previous study [18]. Weight loss was determined by a “yes” response to the question, “Have you lost 2–3 kg or more in the past six months?”: A supporting information, “This does not include a medical doctor’s guidance or intentional diet for health” was provided based on the Kihon Checklist questionnaire [22].

### Social participation

Social participation was assessed using a self-administered questionnaire and classified into five types: volunteer groups, sports groups/clubs, neighborhood associations, religious organizations/groups, and community elderly salons [23]. Participants reported their frequency of participation in each type of organization by choosing one of the following response options: four times or more per week, two to three times per week, once a week, one to three times per month, several times per year, and never. The response was categorized as “yes” if the participants answered “several times per year or more” and “no” if they answered “never” [24]. Involvement in various types of social activities was counted and classified from 5 (participation in all) to 0 (no participation). The number of social participation activities was used as a continuous variable [15]. In addition, we used each type of social participation as a binary variable (yes or no) in order to assess the association between physical prefrailty and types of social participation.

### Covariates

Data on gender (men or women), age (≥75 or <75 years), current smoking habit (yes or no), medication-taking, educational attainment, working status, and living arrangement were obtained using a questionnaire. Medication-taking for the treatment of hypertension, high cholesterol level, or diabetes was categorized into three groups (no medication, 1 medicine, and 2–3 medicines). Educational attainment was defined as the highest education completed and was classified into three categories (< 10 years:–junior high school; 10–12 years:–high school; ≥ 13 years:–junior college/college of technology; and higher education). Working status was categorized based on a yes or no answer. Living arrangement was classified as single household (living alone) or non-single household (living with others); single household was defined as living alone based on marital status (no spouse or children). From the obtained data, body mass index (BMI) was calculated by dividing the body weight by the squared height (kg/m^2^).

### Statistical analysis

Missing information about independent variables, which ranged from 3.1% to 26.0% (S1 Table), was processed using multiple imputations under the missing at random assumption [25]. Each imputation was based on regression models of the analyzed variables. The 10 imputed datasets were analyzed independently and combined for inference, accounting for the variability of imputation [25]. Participants’ characteristics were described by frailty status. The number in each frailty status was estimated as all imputed binary variables to ranging between 0 and 1 rather than rounding values [26]. Between-group differences were determined using a χ2 test for categorical variables and the analysis of variance or Kruskal-Wallis test for continuous data. After excluding participants with frailty (n = 28), in the cross-sectional analyses (n = 588), binominal logistic regression analyses were performed to estimate the odds ratios (OR) and 95% confidence intervals (CI) for having prefrailty by the number or types of social participation activities. Independent variables were not adjusted for in Model 1, and Model 2 included gender (reference category: men), age (reference category: < 75 years), BMI (continuous variable), smoking (reference category: no), medication-taking (reference category: no), educational attainment (reference category: < 10 years), working status (reference category: yes), and living arrangement (reference category: living with others). None of the variables had correlations of sufficient strength to indicate multicollinearity (r < 0.39, S2 Table). Statistical analyses were performed using SPSS version 23 (IBM Corp., Armonk, NY, USA). All p-values for statistical tests were two-tailed, and values < 0.05 were regarded as statistically significant.

## Results

A total of 616 older adults (232 men and 384 women) participated in this study. Of them, 4.5% had frailty (men: 3.4%; women: 5.2%), and 44.3% had prefrailty (men: 41.8%; women: 45.8%) as shown in S1 Table. Table 1 shows the prevalence of frailty by the number and types of social participation activities. The mean (standard deviation) number of social participation activities by frailty status was 2.3 (1.5) in robust participants, 1.9 (1.4) in prefrail participants, and 1.7 (1.4) in frail participants, respectively. As for the types of social participation activities, prevalence rates for prefrailty (51.2%) and frailty (8.7%) were the highest for those who did not participate in their neighborhood associations.

**Table 1:** Participant characteristics by frailty status based on data after multiple imputation.

Variables	Total	Robust	Prefrailty	Frailty	p value*
Number of social participation activities, mean (SD)	2.1 (1.5)	2.3 (1.5)	1.9 (1.4)	1.7 (1.4)	**0.02**
Type of social participation					
Volunteer groups					
No, n (%)	374.2 (60.7)	183.4 (49.0)	169.8 (45.4)	21.0 (5.6)	0.22
Yes, n (%)	241.8 (39.3)	131.6 (54.4)	103.2 (42.7)	7.0 (2.9)	
Sports clubs/groups					
No, n (%)	438.5 (71.2)	201.1 (45.9)	213.7 (48.7)	23.7 (5.4)	**< 0.001**
Yes, n (%)	177.5 (28.8)	113.9 (64.2)	59.3 (33.4)	4.3 (2.4)	
Neighborhood associations					
No, n (%)	220.3 (35.8)	88.5 (40.2)	112.7 (51.2)	19.1 (8.7)	**< 0.001**
Yes, n (%)	395.7 (64.2)	226.5 (57.2)	160.3 (40.5)	8.9 (2.2)	
Religious organizations/groups					
No, n (%)	322.3 (52.3)	163.3 (50.7)	144.7 (44.9)	14.3 (4.4)	0.82
Yes, n (%)	293.7 (47.7)	151.7 (51.7)	128.3 (43.7)	13.7 (4.7)	
Community elderly salons					
No, n (%)	424.7 (68.9)	216.4 (51.0)	195.1 (45.9)	13.2 (3.1)	0.06
Yes, n (%)	191.3 (31.1)	98.6 (51.5)	77.9 (40.7)	14.8 (7.7)	
Gender					
Men, n (%)	232	127 (54.7)	97 (41.8)	8 (3.4)	0.29
Women, n (%)	384	188 (49.0)	176 (45.8)	20 (5.2)	
Age					
≥ 75 years, n (%)	310	147 (47.4)	138 (44.5)	25 (8.1)	**< 0.001**
< 75 years, n (%)	306	168 (54.9)	135 (44.1)	3 (1.0)	
Body mass index, kg/m^2,^ mean (SD)	23.0 (3.1)	22.8 (3.2)	23.1 (3.0)	22.5 (3.6)	0.35
Smoking					
Yes, n (%)	36	18 (50.0)	17 (47.2)	1 (2.8)	0.84
No, n (%)	580	297 (51.2)	256 (44.1)	27 (4.7)	
Medication					
2–3 medicines, n (%)	128	54 (42.2)	66 (51.6)	8 (6.3)	0.11
1 medicine, n (%)	236	125 (53.0)	98 (41.5)	13 (5.5)	
No, n (%)	252	136 (54.0)	109 (43.3)	7 (2.8)	
Educational attainment					
< 10 years, n (%)	204.7	91.9 (44.9)	96.6 (47.2)	16.2 (7.9)	**0.03**
10–12 years, n (%)	225.0	119.7 (53.2)	97.7 (43.4)	7.6 (3.4)	
≥ 13 years, n (%)	186.3	103.4 (55.5)	78.7 (42.2)	4.2 (2.3)	
Working status					
No, n (%)	454.7	228.5 (50.3)	202.9 (44.6)	23.3 (5.1)	0.39
Yes, n (%)	161.3	86.5 (53.6)	70.1 (43.5)	4.7 (2.9)	
Living arrangement					
Lives alone, n (%)	113.2	52.1 (46.0)	56.1 (49.6)	5.0 (4.4)	0.42
Lives with others, n (%)	502.8	262.9 (52.3)	216.9 (43.1)	23 (4.6)	

SD, standard deviation.

*Statistical significance of the differences between groups was determined using the χ2 test for categorical data and the analysis of variance or Kruskal-Wallis test for continuous data. Values in boldface show significance (p < 0.05).

The number (decimal) in each frailty status was shown using data after multiple imputations according to Allison [26].

Table 2 shows the associations between the number of social participation activities and prefrailty. The binominal logistic regression analysis showed that the number of social participation activities was significantly associated with lower odds of prefrailty (OR: 0.83, 95% CI: 0.74–0.94) in Model 2.

**Table 2:** Associations between number of social participation activities and prefrailty among Japanese older adults (n = 588).

	Model 1*	Model 2^†^
	Prefrailty	Prefrailty
	OR (95% CI)	OR (95% CI)
Number of social participation activities	**0.84 (0.75**–**0.95)**	**0.83 (0.74**–**0.94)**
Demographic characteristics		
Gender, women		1.16 (0.81–1.68)
Age, ≥ 75 years		1.11 (0.75–1.62)
Body mass index		1.03 (0.97–1.09)
Smoking, yes		1.18 (0.56–2.50)
Medication-taking		
1 medicine		1.00 (0.68–1.48)
2–3 medicines		1.52 (0.95–2.42)
Educational attainment		
10–12 years		0.83 (0.54–1.27)
≥ 13 years, n (%)		0.82 (0.52–1.30)
Working status, no		1.08 (0.66–1.76)
Living arrangement, lives alone		1.31 (0.83–2.05)

Social participation was analyzed using binomial logistic regression. Values in boldface indicate significance (p < 0.05).

OR, odds ratio; CI, confidence interval.

*Model 1: crude model.

^†^Model 2: gender, age, body mass index, smoking status, medication-taking, educational attainment, working status, and living arrangement were adjusted for.

Table 3 shows the associations between the types of social participation and prefrailty. The binominal logistic regression analysis showed that participation in sports clubs/groups was significantly associated with prefrailty (OR: 0.47, 95% CI: 0.31–0.73) in Model 2. Participation in neighborhood associations was also significantly associated with prefrailty (OR: 0.57, 95% CI: 0.37–0.86) in Model 2. However, participation in volunteer groups, religious organizations/groups, and community elderly salons was not associated with prefrailty.

**Table 3:** Associations between the types of social participation and prefrailty among Japanese older adults (n = 588).

	Model 1*	Model 2^†^
	Prefrailty	Prefrailty
	OR (95% CI)	OR (95% CI)
Type of social participation^‡^		
Volunteer groups	1.10 (0.74–1.65)	1.12 (0.74–1.69)
Sports clubs/groups	**0.50 (0.33**–**0.75)**	**0.47 (0.31**–**0.73)**
Neighborhood associations	**0.52 (0.35**–**0.78)**	**0.57 (0.37**–**0.86)**
Religious organizations/groups	1.34 (0.91–1.99)	1.35 (0.89–2.05)
Community elderly salon	0.96 (0.65–1.42)	0.79 (0.52–1.20)
Demographic characteristics		
Gender, women		1.27 (0.86–1.88)
Age, ≥ 75 years		1.20 (0.80–1.79)
Body mass index		1.03 (0.97–1.09)
Smoking		
Yes		1.10 (0.51–2.35)
Medication-taking		
1 medicine		1.00 (0.67–1.48)
2–3 medicines		1.46 (0.90–2.36)
Educational attainment		
10–12 years		0.91 (0.58–1.42)
≥ 13 years, n (%)		0.93 (0.57–1.52)
Working status, no		1.06 (0.64–1.78)
Living arrangement, lives alone		1.27 (0.80–2.01)

Social participation was analyzed using binomial logistic regression. Values in boldface indicate significance (p<0.05).

OR, odds ratio; CI, confidence interval.

*Model 1: crude model.

^†^Model 2: gender, age, body mass index, smoking status, medication-taking, educational attainment, working status, and living arrangement were adjusted for.

^‡^Reference: no social participation.

## Discussion

This study is the first to examine the association between the number or types of social participation activities and physical prefrailty in older Japanese adults. Two major findings were obtained. First, regarding the number of social participation activities, an increase by one was associated with lower odds of prefrailty. Second, older adults participating in sports clubs and neighborhood associations had significantly lower odds of prefrailty. Our findings suggest that focusing on the number and types of social participation activities may contribute to preventing physical prefrailty among older Japanese adults.

Our findings are consistent with some previous studies. A cross-sectional study among older adults in Taiwan reported associations between physical frailty based on the Fried index and the number of social activities including leisure, religious activities, visiting friends and relatives, and chatting with neighbors [15]. The number of social activities, considered a continuous variable, was negatively associated with prefrailty and frailty. Ye et al.’s study used a total score based on the frequency of participation in any of the eight types of activities in Chinese older adults [16]. The increasing score of the frequency of social participation activities was negatively associated with frailty. Abe et al.’s study used the Kaigo-Yobo Checklist to examine whether frailty was associated with social participation activities among community-dwelling older adults in Japan [13]. Their study examined social participation across three levels of frequency (regular, irregular, and non-participation) in any of the eight types of activities. Those who regularly participated in social activities reported improvements in their frailty status. A relationship between social participation and physical health outcomes has also been reported in some previous studies. Kanamori et al.’s four-year follow-up study on older Japanese adults found that the number and types of social participation activities were associated with functional disability [27]. The previously mentioned study showed that participation in three or more organizations was associated with lower incidence of disability. Moreover, as for the type of social participation activity, participation in the local community, including neighborhood associations and sports clubs/groups, was associated with a lower likelihood of disability. Further, a three-year follow-up study examined whether the number and types of social participation activities were associated with instrumental activities of daily living among community-dwelling older men and women [28]. The study found that participation in three or more activities was associated with lower odds of decline in instrumental activities of daily living for both men and women in the age-adjusted model. Moreover, participation in neighborhood community associations was associated with a lower risk of decline in instrumental activities of daily living among older women.

The mechanisms underlying the association between social participation and frailty among older adults are likely complex [29]. We speculated about the mechanism underpinning the relationship between social participation and physical prefrailty based on a previous finding, according to which social participation provides opportunities for older adults to access various social support networks [30]. For example, it might be easier for older adults to access health information at various social events. Moreover, a high frequency of social participation activities is positively associated with physical activity, which may contribute to improving motor function and mental health [31–33]. Meanwhile, for the association between participation in sports clubs/groups and prefrailty, systematic reviews reported the benefits of physical exercise training for frail older adults [34, 35]. This may explain the inverse association between participation in sports clubs/groups and prefrailty.

Participation in community salons showed a positive trend with frailty, as shown in Table 1 (p = 0.06), although the association was not significant in any adjusted model. In 2017, the Japanese government launched the “community elderly salon” program in neighborhood communities as a national long-term care strategy [36]. Therefore, the positive relationship might be explained by the fact that frail older adults in the community were preferentially encouraged to participate. In the long-term, participation in community salons may reduce the prevalence of frailty [23]. Our results suggest that future longitudinal studies are required to ascertain the details of the positive or negative relationship between participation in community elderly salons and frailty.

Regarding the relationship between demographic characteristics and frailty, only age showed a positive association, as depicted in Table 1. A meta-analysis of studies conducted among Japanese adults showed that the prevalence of frailty was significantly higher among older adults [8]. Our results are in keeping with this finding. Thus, in a super-aging society like Japan, it is important to prevent frailty as early as possible.

This study has several limitations. First, we used a cross-sectional design, which precludes the possibility of a causal inference between social participation and frailty. Older adults with poor physical functioning may not participate in social groups, suggesting that frailty might be a reason for the lack of social participation. Second, the participants were recruited from multiple centers and an annual health examination, which may have resulted in a selection bias. As the small sample caused a low statistical power, our results might underestimate the associations. Third, this study used a modified version of the frailty phenotype with J-CHS criteria to assess frailty in Japanese individuals; a previous review has reported that a modification of the frailty phenotype affects its classification and predictive ability [37]. Fourth, although social participation, physical activity, exhaustion, and weight loss associated with frailty were measured using self-report questionnaires based on the J-CHS [18], the relationship between social participation and frailty may have been over- or under-estimated because of a response bias. Fifth, although the item for measuring social participation was used in a previous study in Japan [27], it did not measure participant activities and the size of the unit in each social participation group. Moreover, type of social participation did not include professional organizations, politics, and cultural activities. Additionally, participants’ involvement and the activity’s content or variety (e.g., recreational walking at sports clubs, group conversations at neighborhood associations, cleaning streets with volunteer groups) may also affect older adults’ mental or physical health. Finally, we could not control for the effects of unmeasured factors, such as musculoskeletal disorders, cognitive status, and depression, on the relationship between social participation and prefrailty [33, 38–41].

## Conclusions

This study found that a higher number of social participation activities was associated with lower odds of prefrailty among older adults living in a rural area of Japan. Further, participation in sports clubs/groups or neighborhood associations was associated with lower odds of prefrailty. Our findings suggest that social participation may be important to prevent physical prefrailty. However, further intervention-based longitudinal studies are needed to verify whether social participation has preventative effects on prefrailty.

## Supporting information

S1 Table Participant characteristics. SD, standard deviation.
(DOCX)

S2 Table Correlation coefficient of independent variables. *p < 0.05, **p < 0.01.
(DOCX)

S1 Data (XLSX)

## References

[1] Clegg A, Young J, Iliffe S, Rikkert MO, Rockwood K. Frailty in elderly people. Lancet. 2013;381(9868):752–762. 10.1016/s0140-6736(12)62167-9 23395245 PMC4098658

[2] Kojima G, Iliffe S, Jivraj S, Walters K. Association between frailty and quality of life among community-dwelling older people: a systematic review and meta-analysis. J Epidemiol Community Health. 2016;70(7):716–721. 10.1136/jech-2015-206717 26783304

[3] Kojima G. Frailty as a predictor of disabilities among community-dwelling older people: a systematic review and meta-analysis. Disabil Rehabil. 2017;39(19):1897–1908. 10.1080/09638288.2016.1212282 27558741

[4] Kojima G, Iliffe S, Walters K. Frailty index as a predictor of mortality: a systematic review and meta-analysis. Age Ageing. 2018;47(2):193–200. 10.1093/ageing/afx162 29040347

[5] Fried LP, Tangen CM, Walston J, Newman AB, Hirsch C, Gottdiener J, et al Frailty in older adults: evidence for a phenotype. J Gerontol A Biol Sci Med Sci. 2001;56(3):M146–M156. 10.1093/gerona/56.3.m146 11253156

[6] Choi J, Ahn A, Kim S, Won CW. Global prevalence of physical frailty by Fried’s criteria in community-dwelling elderly with national population-based surveys. J Am Med Dir Assoc. 2015;16(7):548–550. 10.1016/j.jamda.2015.02.004 25783624

[7] Collard RM, Boter H, Schoevers RA, Oude Voshaar RC. Prevalence of frailty in community-dwelling older persons: a systematic review. J Am Geriatr Soc. 2012; 60(8): 1487–1492. 22881367 10.1111/j.1532-5415.2012.04054.x

[8] Kojima G, Iliffe S, Taniguchi Y, Shimada H, Rakugi H, Walters K. Prevalence of frailty in Japan: a systematic review and meta-analysis. J Epidemiol. 2017;27(8):347–353. 10.1016/j.je.2016.09.008 28142044 PMC5549151

[9] Hoogendijk EO, Afilalo J, Ensrud KE, Kowal P, Onder G, Fried LP. Frailty: implications for clinical practice and public health. Lancet. 2019;394(10206):1365–1375. 10.1016/S0140-6736(19)31786-6 31609228

[10] Bunt S, Steverink N, Olthof J, van der Schans CP, Hobbelen JSM. Social frailty in older adults: a scoping review. Eur J Ageing. 2017;14:323–334. 10.1007/s10433-017-0414-7 28936141 PMC5587459

[11] Duppen D, Van der Elst MCJ, Dury S, Lambotte D, De Donder L. The social environment’s relationship with frailty: evidence from existing studies. J Appl Gerontol. 2019;38(1):3–26. 10.1177/0733464816688310 28380715

[12] Etman A, Kamphuis CB, van der Cammen TJ, Burdorf A, van Lenthe FJ. Do lifestyle, health and social participation mediate educational inequalities in frailty worsening? Eur J Public Health. 2015;25(2):345–350. 10.1093/eurpub/cku093 25061232 PMC4447813

[13] Abe T, Nofuji Y, Seino S, Murayama H, Yoshida Y, Tanigaki T, et al Healthy lifestyle behaviors and transitions in frailty status among independent community-dwelling older adults: the Yabu Cohort Study. Maturitas. 2020;136:54–59. 10.1016/j.maturitas.2020.04.007 32386667

[14] Woo J, Goggins W, Sham A, Ho SC. Social determinants of frailty, Gerontology. 2005;51(6):402–408. 10.1159/000088705 16299422

[15] Chen LJ, Chen CY, Lue BH, Tseng MY, Wu SC. Prevalence and associated factors of frailty among elderly people in Taiwan. Int J Gerontol. 2014;8(3):114–119. 10.1016/j.ijge.2013.12.002

[16] Ye B, Gao J, Fu H. Associations between lifestyle, physical and social environments and frailty among Chinese older people: a multilevel analysis. BMC Geriatr. 2018;18(1):314 10.1186/s12877-018-0982-1 30547760 PMC6295038

[17] Vogelsang EM. Older adult social participation and its relationship with health: rural-urban differences. Health Place. 2016;42:111–119. 10.1016/j.healthplace.2016.09.010 27755999 PMC5116414

[18] Satake S, Shimada H, Yamada M, Kim H, Yoshida H, Gondo Y, et al Prevalence of frailty among community-dwellers and outpatients in Japan as defined by the Japanese version of the Cardiovascular Health Study criteria. Geriatr Gerontol Int. 2017;17(12):2629–2634. 29265757 10.1111/ggi.13129

[19] Takayanagi N, Sudo M, Fujii M, Sakai H, Morimoto K, Tomisaki M, et al Foot pressure analysis of gait pattern in older Japanese females requiring different personal care support levels. J Phys Ther Sci. 2018;30(3):461–466. 10.1589/jpts.30.461 29581672 PMC5857459

[20] Chen LK, Woo J, Assantachai P, Auyeung TW, Chou MY, Iijima K, et al Asian Working Group for Sarcopenia: 2019 consensus update on sarcopenia diagnosis and treatment. J Am Med Dir Assoc. 2020;21(3):300–307.e2. 10.1016/j.jamda.2019.12.012 32033882

[21] Chen LK, Liu LK, Woo J, Assantachai P, Auyeung TW, Bahyah KS, et al Sarcopenia in Asia: consensus report of the Asian Working Group for Sarcopenia. J Am Med Dir Assoc. 2014;15(2):95–101. 10.1016/j.jamda.2013.11.025 24461239

[22] Satake S, Senda K, Hong Y-J, Miura H, Endo H, Sakurai T, et al Validity of the Kihon Checklist for assessing frailty status. Geriatr Gerontol Int. 2016;16(6):709–715. 10.1111/ggi.12543 26171645

[23] Nakagawa K, Kawachi I. What types of activities increase participation in community “salons”? Soc Sci Med. 2019;238:112484 10.1016/j.socscimed.2019.112484 31421351

[24] Ide K, Tsuji T, Kanamori S, Jeong S, Nagamine Y, Kondo K. Social participation and functional decline: a comparative study of rural and urban older people, using Japan Gerontological Evaluation Study longitudinal data. Int J Environ Res Public Health. 2020;17(2):617 10.3390/ijerph17020617 PMC7013913 31963645

[25] Barnard J, Meng XL. Applications of multiple imputation in medical studies: from AIDS to NHANES. Stat Methods Med Res. 1999;8(1):17–36. 10.1177/096228029900800103 10347858

[26] Allison PD. Imputation of categorical variables with PROC MI [Internet]. In: 30th Annual SAS Users Group International Conference (SUGI 30); 2005 Apr 10–13; Philadelphia, Pennsylvania. Paper 113–30. [cited 26 Oct 2020]. https://support.sas.com/resources/papers/proceedings/proceedings/sugi30/113-30.pdf

[27] Kanamori S, Kai Y, Aida J, Kondo K, Kawachi I, Hirai H, et al Social participation and the prevention of functional disability in older Japanese: the JAGES cohort study. PLoS One. 2014;9(6):e99638 10.1371/journal.pone.0099638 24923270 PMC4055714

[28] Tomioka K, Kurumatani N, Hosoi H. Association between social participation and 3-year change in instrumental activities of daily living in community-dwelling elderly adults. J Am Geriatr Soc. 2017;65(1):107–113. 10.1111/jgs.14447 27673582

[29] Serrat R, Scharf T, Villar F, Gómez C. Fifty-five years of research into older people’s civic participation: recent trends, future directions. Gerontologist. 2020;60(1):e38–e51. 10.1093/geront/gnz021 30889249 PMC12774630

[30] Kawachi I, Berkman LF. Social ties and mental health. J Urban Health. 2001;78:458–467. 10.1093/jurban/78.3.458 11564849 PMC3455910

[31] Kikuchi H, Inoue S, Fukushima N, Takamiya T, Odagiri Y, Ohya Y, et al Social participation among older adults not engaged in full- or part-time work is associated with more physical activity and less sedentary time. Geriatr Gerontol Int. 2017;17(11):1921–1927. 10.1111/ggi.12995 28230301

[32] James BD, Boyle PA, Buchman AS, Bennett DA. Relation of late-life social activity with incident disability among community-dwelling older adults. J Gerontol A Biol Sci Med Sci. 2011;66A(4):467–473. 10.1093/gerona/glq231 PMC3055280 21300745

[33] Hamano T, Fujisawa Y, Ishida Y, Subramanian SV, Kawachi I, Shiwaku K. Social capital and mental health in Japan: a multilevel analysis. PLoS One. 2010;5(10):e13214 10.1371/journal.pone.0013214 20949091 PMC2950857

[34] de Labra C, Guimaraes-Pinheiro C, Maseda A, Lorenzo T, Millán-Calenti JC. Effects of physical exercise interventions in frail older adults: a systematic review of randomized controlled trials. BMC Geriatr. 2015;15:154 10.1186/s12877-015-0155-4 26626157 PMC4667405

[35] Chin APMJ, van Uffelen JG, Riphagen I, van Mechelen W. The functional effects of physical exercise training in frail older people: a systematic review. Sports Med. 2008;38(9):781–793. 10.2165/00007256-200838090-00006 18712944

[36] Ministry of Health, Labour and Welfare of Japan. Establishing the community-based integrated care system [Internet]. Tokyo: The Mnistry; 2017. [cited 2020 Aug 5]. https://www.mhlw.go.jp/english/policy/care-welfare/care-welfare-elderly/dl/establish_e.pdf.

[37] Theou O, Cann L, Blodgett J, Wallace LM, Brothers TD, Rockwood K. Modifications to the frailty phenotype criteria: systematic review of the current literature and investigation of 262 frailty phenotypes in the Survey of Health, Ageing, and Retirement in Europe. Ageing Res Rev. 2015;21:78–94. 10.1016/j.arr.2015.04.001 25846451

[38] Perruccio AV, Yip C, Power JD, Canizares M, Gignac MA, Badley EM. Understanding the association between osteoarthritis and social participation: a CLSA population-based study. Arthritis Care Res. 2020 10.1002/acr.24366 32598513

[39] Castell MV, van der Pas S, Otero A, Siviero P, Dennison E, Denkinger M, et al Osteoarthritis and frailty in elderly individuals across six European countries: results from the European Project on OSteoArthritis (EPOSA). BMC Musculoskelet Disord. 2015;16:359 10.1186/s12891-015-0807-8 26578262 PMC4650343

[40] Ito T, Okuyama K, Abe T, Takeda M, Hamano T, Nakano K, et al Relationship between individual social capital and cognitive function among older adults by gender: a cross-sectional study. Int J Environ Res Public Health. 2019;16(12):2142 10.3390/ijerph16122142 PMC6616497 31212979

[41] HajekA, BrettschneiderC, PosseltT, LangeC, MamoneS, WieseB, et al Predictors of frailty in old age–results of a longitudinal study. J Nutr Health Aging. 2016;20:952–957. 10.1007/s12603-015-0634-527791226

