# Supplementary material for: Social participation and physical prefrailty in older Japanese adults: The Shimane CoHRE study
Source: PLoS One. 2020 Dec 16;15(12):e0243548. doi: 10.1371/journal.pone.0243548 (PMC7743931; doi:10.1371/journal.pone.0243548)
Supplement: S1 Table — SD, standard deviation. (DOCX) [file pone.0243548.s001.docx]

| **S1 Table. Participant Characteristics.** | | | | |
| --- | --- | --- | --- | --- |
| Variables | | Total, N = 616 | Men, n = 232 | Women, n = 384 |
| Frailty | |  |  |  |
|  | Robust, n (%) | 315 (51.1) | 127 (54.7) | 188 (49.0) |
|  | Prefrailty, n (%) | 273 (44.3) | 97 (41.8) | 176 (45.8) |
|  | Frailty, n (%) | 28 (4.5) | 8 (3.4) | 20 (5.2) |
| Age | |  |  |  |
|  | ≥ 75 years, n (%) | 310 (50.3) | 118 (50.9) | 192 (50.0) |
|  | < 75 years, n (%) | 306 (49.7) | 114 (49.1) | 192 (50.0) |
| Body mass index, kg/m^2^ (SD) | | 23.0 (3.1) | 23.2 (2.9) | 22.8 (3.2) |
| Smoking | |  |  |  |
|  | Yes, n (%) | 36 (5.8) | 30 (12.9) | 6 (1.6) |
|  | No, n (%) | 580 (94.2) | 202 (87.1) | 378 (98.4) |
| Medication | |  |  |  |
|  | 2–3 medicines, n (%) | 128 (20.8) | 36 (15.5) | 92 (24.0) |
|  | 1 medicine, n (%) | 236 (38.3) | 95 (40.9) | 141 (36.7) |
|  | No, n (%) | 252 (40.9) | 101 (43.5) | 151 (39.3) |
| Educational attainment | |  |  |  |
|  | < 10 years, n (%) | 196 (29.2) | 61 (26.3) | 135 (35.2) |
|  | 10–12 years, n (%) | 216 (35.1) | 87 (37.5) | 129 (33.6) |
|  | ≥ 13 years, n (%) | 180 (29.2) | 75 (32.3) | 105 (27.3) |
|  | Missing information, n (%) | 24 (3.9) | 9 (3.9) | 15 (3.9) |
| Working status | |  |  |  |
|  | No, n (%) | 337 (54.7) | 123 (53.0) | 214 (55.7) |
|  | Yes, n (%) | 119 (19.3) | 60 (25.9) | 59 (15.4) |
|  | Missing information, n (%) | 160 (26.0) | 49 (21.1) | 111 (28.9) |
| Living arrangement | |  |  |  |
|  | Lives alone, n (%) | 104 (16.9) | 27 (11.6) | 77 (20.1) |
|  | Lives with others, n (%) | 472 (76.6) | 195 (84.1) | 277 (72.1) |
|  | Missing information, n (%) | 40 (6.5) | 10 (4.3) | 30 (7.8) |
| Social participation | |  |  |  |
| Volunteer groups | |  |  |  |
|  | No, n (%) | 345 (56.0) | 139 (59.9) | 206 (53.6) |
|  | Yes, n (%) | 211 (34.3) | 80 (34.5) | 131 (34.1) |
|  | Missing information, n (%) | 60 (9.7) | 13 (5.6) | 47 (12.2) |
| Sports clubs/groups | |  |  |  |
|  | No, n (%) | 395 (64.1) | 155 (66.8) | 240 (62.5) |
|  | Yes, n (%) | 155 (25.2) | 61 (26.3) | 94 (24.5) |
|  | Missing information, n (%) | 66 (10.7) | 16 (6.9) | 50 (13.0) |
| Neighborhood associations | |  |  |  |
|  | No, n (%) | 195 (31.7) | 65 (28.0) | 130 (33.9) |
|  | Yes, n (%) | 350 (56.8) | 150 (64.7) | 200 (52.1) |
|  | Missing information, n (%) | 71 (11.5) | 17 (7.3) | 54 (14.1) |
| Religious organizations/groups | |  |  |  |
|  | No, n (%) | 291 (47.2) | 86 (37.1) | 205 (53.4) |
|  | Yes, n (%) | 253 (41.1) | 133 (57.3) | 120 (31.3) |
|  | Missing information, n (%) | 72 (11.7) | 13 (5.6) | 59 (15.4) |
| Community elderly salons | |  |  |  |
|  | No, n (%) | 385 (62.5) | 171 (73.7) | 214 (55.7) |
|  | Yes, n (%) | 168 (27.3) | 38 (16.4) | 130 (33.9) |
|  | Missing information, n (%) | 63 (10.2) | 23 (9.9) | 40 (10.4) |

SD, standard deviation
