# Supplementary material for: Social participation and physical prefrailty in older Japanese adults: The Shimane CoHRE study
Source: PLoS One. 2020 Dec 16;15(12):e0243548. doi: 10.1371/journal.pone.0243548 (PMC7743931; doi:10.1371/journal.pone.0243548)
Supplement: S2 Table — *p < 0.05, **p < 0.01. (DOCX) [file pone.0243548.s002.docx]

| **S2 Table. Correlation Coefficient of Independent Variables.** | | | | | | | | | | | | | | |
| --- | --- | --- | --- | --- | --- | --- | --- | --- | --- | --- | --- | --- | --- | --- |
| Variables | Number of social participation activities | Volunteer groups | Sports clubs/groups | Neighborhood associations | Religious organizations/groups | Community elderly salons | Gender | Age | Body mass index | Smoking | Medication | Educational attainment | Working status | Living arrangement |
| Number of social participation activities | 1.00 |  |  |  |  |  |  |  |  |  |  |  |  |  |
| Volunteer groups | 0.68** | 1.00 |  |  |  |  |  |  |  |  |  |  |  |  |
| Sports clubs/groups | 0.53** | 0.23** | 1.00 |  |  |  |  |  |  |  |  |  |  |  |
| Neighborhood associations | 0.65** | 0.31** | 0.15** | 1.00 |  |  |  |  |  |  |  |  |  |  |
| Religious organizations/groups | 0.67** | 0.27** | 0.18** | 0.39** | 1.00 |  |  |  |  |  |  |  |  |  |
| Community elderly salons | 0.50** | 0.26** | 0.15** | 0.09 | 0.13** | 1.00 |  |  |  |  |  |  |  |  |
| Gender | 0.02 | -0.04 | -0.01 | 0.08 | 0.20** | -0.21** | 1.00 |  |  |  |  |  |  |  |
| Age | -0.08 | 0.00 | -0.14** | 0.08 | -0.01 | -0.21** | -0.02 | 1.00 |  |  |  |  |  |  |
| Body mass index | 0.00 | 0.02 | -0.01 | -0.07 | 0.00 | 0.08 | 0.08 | -0.03 | 1.00 |  |  |  |  |  |
| Smoking | 0.06 | 0.06 | 0.12** | -0.03 | -0.06 | 0.12** | -0.25** | -0.20** | 0.03 | 1.00 |  |  |  |  |
| Medication | -0.12** | -0.02 | -0.07 | 0.00 | -0.10* | -0.18** | 0.06 | 0.22** | -0.19** | -0.14** | 1.00 |  |  |  |
| Educational attainment | -0.08 | -0.07 | -0.06 | -0.18** | 0.01 | 0.07 | -0.08 | -0.22** | 0.09* | 0.03 | -0.06 | 1.00 |  |  |
| Working status | -0.07 | -0.04 | -0.06 | 0.03 | 0.01 | -0.17** | 0.12** | 0.30** | 0.10* | -0.03 | 0.09 | 0.03 | 1.00 |  |
| Living arrangement | -0.06 | -0.01 | -0.01 | -0.01 | -0.06 | -0.09 | 0.12** | 0.08 | 0.02 | 0.07 | 0.02 | -0.06 | 0.05 | 1.00 |
| *p < 0.05, **p < 0.01 | | | | | | | | | | | | | | |
